# Quantitative Analysis of the Effect of Fluorescent Labels on DNA Strand Displacement Reaction

**DOI:** 10.3390/mi15121466

**Published:** 2024-11-30

**Authors:** Masato Toyonari, Kaori Aso, Takashi Nakakuki

**Affiliations:** Department of Intelligent and Control Systems, Faculty of Computer Science and Systems Engineering, Kyushu Institute of Technology, 680-4 Kawazu, Iizuka 820-8502, Fukuoka, Japan; toyonari.masato494@mail.kyutech.jp (M.T.); aso.kaori625@mail.kyutech.jp (K.A.)

**Keywords:** DNA computing, DNA strand displacement reaction, reaction kinetics, reporter molecules

## Abstract

DNA chemical reaction networks can perform complex information processing through careful design of reaction kinetics, which involves the reaction network structure, rate constants, and initial concentrations. The toehold-mediated strand displacement reaction (TMSDR) is a key mechanism in creating DNA circuits, offering a rational design approach by integrating individually designed TMSDRs. Tools such as VisualDSD and NUPACK facilitate the efficient design of these systems by allowing precise tuning of reaction parameters. However, discrepancies between simulated and experimental results can occur, often due to the modification of reporter molecules. Recently, fluorophore dyes and quenchers were found to significantly impact the dynamics of irreversible TMSDRs, altering them by nearly two orders of magnitude. The impact on reaction dynamics varies with the modification site of these reporters. This study examines the mechanisms of reporter modifications affecting reversible TMSDRs, influencing transient and steady-state characteristics. This is crucial for DNA circuit design, which integrates reversible and irreversible TMSDRs. Our findings indicate that modifying fluorescent dye and quencher an appropriate distance apart (e.g., toehold length) can minimize adverse effects on the DNA reaction dynamics while ensuring effective FRET, therefore improving the accuracy of experimental verification for DNA reaction systems.

## 1. Introduction

DNA chemical reaction networks can perform elaborate information processing by appropriately designing the kinetics of the reaction system. The kinetics of the reaction system is defined by the reaction network structure, the reaction rate constants, and the initial concentrations, which are required to design the base sequences of DNA strands involved in the reaction system, based on DNA complementarity [1]. Toehold-mediated strand displacement reactions (TMSDRs) are widely recognized as versatile reaction mechanisms for fabricating DNA circuits [2]. They offer an entire practical framework of the “rational design”, where the entire system is constructed by incorporating individually pre-designed TMSDRs.

Currently, various computer-aided design tools for DNA reaction systems such as VisualDSD [3], NUPACK [4], etc., are available; hence, we can efficiently design the information processing mechanism rationally by assembling the reaction network structure, tuning the reaction rate constants, and determining initial concentrations. Notably, due to the availability of reliable estimates for TMSDR rate constants [5], the predicted reaction dynamics in silico under “ideal” experimental conditions closely align with experimental observations. Here, “dynamics” refers to two aspects: transient characteristics, which describe the time evolution profile of concentration, and steady-state characteristics, which represent the concentration balance after the reaction. Meanwhile, under some circumstances, simulation results fail to predict the experimental results qualitatively or quantitatively, even if a DNA reaction system in which base sequences of all DNA strands are designed perfectly is carefully experimented with under target temperature and appropriate buffer conditions. Among various factors possibly behind this mismatch issue, the most common factor encountered is the change in reaction dynamics due to modification of reporter molecules [6]. Recently, Li et al. evaluated the effect of fluorophore dyes and quenchers such as FAM, Cy5, and BHQ1 on the reaction dynamics of irreversible TMSDRs, indicating that these modifications significantly modulate the dynamics by almost two orders of magnitude [7]. Furthermore, the effect on dynamics was complex wherein the transient characteristics changed depending on the modification location of the fluorophore dyes and quenchers. Importantly, measuring the dynamics of reaction systems using these reporter molecules (or probes) is a common method widely utilized in DNA reaction system design. Even if a DNA reaction system is designed to possess the desired dynamics, verifying the validity of the design becomes challenging when the modification of reporter molecules alters the reaction rate constant during the verification experiment.

In this study, we investigated the effect of modification of the reporter molecules on the reaction dynamics for mainly reversible TMSDRs (or toehold-mediated strand exchange reactions), which were not addressed in the previous study. For the irreversible TMSDRs in the previous study [7], the effect of the reporter modification was only on transient characteristics. In contrast, for the reversible version, it appeared in both transient and steady-state characteristics. In the design of DNA circuits that perform complex information processing in the field of DNA computing, identifying ahead how the reporter modifications for measurements affect the reaction dynamics is crucial because both reversible and irreversible TMSDRs are integrated in the design of reaction systems [8,9,10,11,12]. Here, we demonstrate that a reporter modification in which the fluorophore dye and quencher are modified an appropriate distance apart is a suitable method that can reduce the detrimental effect on the dynamics of the DNA reaction system while simultaneously expecting the effectiveness of FRET (fluorescence resonance energy transfer).

## 2. Materials and Methods

### 2.1. Two Variations of TMSDRs

**Figure 1:**
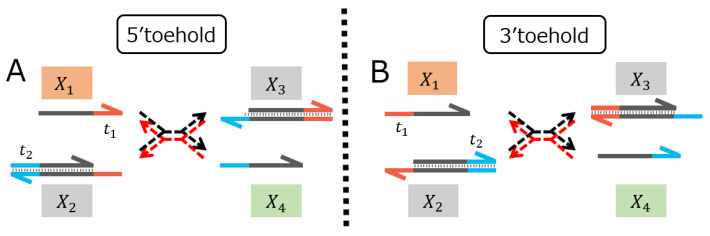
Two variations of the reversible TMSDRs. Arrows indicate single-stranded DNAs, oriented arrows indicate double-stranded DNAs, where an arrow-head indicates the 3′ end, and an arrow-end indicates the 5′ end. The domains $t_{1}$ and $t_{2}$ denote toeholds. Dotted arrows indicate reactions. (**A**) Reversible TMSD with the toehold $t_{1}$ allocated at the 5′ end (5′ toehold) (**B**) Reversible TMSD with the toehold $t_{1}$ allocated at the 3′ end (3′ toehold).

The TMSDR shown in Figure 1 is a reaction mechanism in which the single-stranded DNA (denoted by $X_{1}$) associates with the double-stranded DNA (denoted by $X_{2}$), based on the toehold domain ($t_{1}$), to induce the double-stranded DNA (denoted by $X_{3}$) and the single-stranded DNA (denoted by $X_{4}$), where the toehold domains ($t_{1}$ and $t_{2}$) play a critical role in controlling the reaction rates in the TMSDRs [5]. TMSDRs can be reversible by the structure of strand $X_{2}$. Moreover, the toehold domain on strand $X_{2}$ can be allocated either at the 5′ end (Figure 1A) or at the 3′ end (Figure 1B), implying that two variations can occur for designing the reversible TMSDRs. For the sake of simplicity of notation, we refer to the toehold allocated in the 5′ or 3′ ends of strand $X_{2}$ by “5′ toehold” and “3′ toehold”, respectively. The mathematical models of the reversible TMSDRs, which are employed for parameter estimation of the reaction rate constants, are provided in Supporting Information Text S1.

### 2.2. Base Sequences of Oligo-DNAs

Base sequences of oligo-DNAs corresponding to strands $X_{1}$, $X_{2}$, $X_{3}$, and $X_{4}$ used in the experiments were designed both to reduce undesirable secondary structure and to have approximately the same thermodynamic stability between strand $X_{2}$ (before reactions) and $X_{3}$ (after reactions) while using NUPACK [4]. In all experiments, typical specifications with a 6-mer toehold and 20-mer recognition domains were employed based on [5]. Base sequences and the corresponding NUPACK predictions are provided in Supporting Information Texts S2 and S3.

### 2.3. Preparation for Experiment

All DNA oligonucleotides (Fasmac Co., Ltd., Atsugi, Japan) were suspended in TE buffer (10 mM Tris·HCl pH balanced to 8.0, 1 mM EDTA-2Na, NIPPON GENE Co., Ltd., Tokyo, Japan) at 100 μM and stored at 4 °C. Immediately before the experiments, 1 M ${\mathrm{MgCl}}_{2}$ (NIPPON GENE Co., Ltd.) was added to the samples at a ratio of 1:79, resulting in a final ${\mathrm{MgCl}}_{2}$ concentration of 12.5 mM. The experiments were performed at 25 ± 0.5 °C with temperature control. All double-stranded gates were prepared at a concentration of 5 μM by annealing in a thermal cycler (ASTEC Co., Ltd., Fukuoka, Japan) in TE/${\mathrm{Mg}}^{2+}$ buffer. Annealing was performed by heating up to 95 °C, and maintaining for 15 min, then slowly cooling down to 20 °C at the rate of 1 °C/min according to the protocol given by [13]. In this study, we mainly followed the standard experimental protocol [11], which has been widely employed in many studies.

### 2.4. Fluorescence Measurement

**Figure 2:**
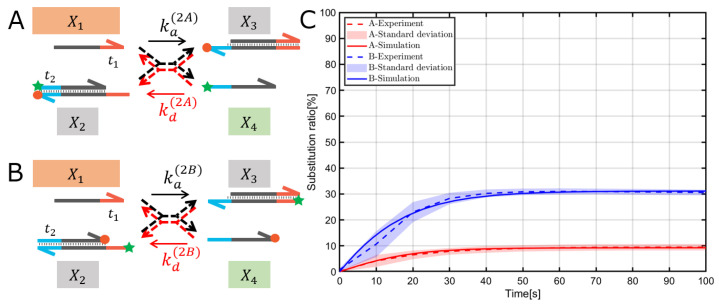
Reversible TMSDRs with the 5′ toehold case: The TMSDRs with the symmetric modification (**A**) and asymmetric modification (**B**) were designed where the star and round shapes denote FAM and BHQ1, respectively. Reaction rate constants of the forward and backward reactions are denoted by $k_{a}^{\left(*\right)}$ and $k_{d}^{\left(*\right)}$ (${\mathrm{nM}}^{-1}s^{-1}$), respectively. (**C**) Experimental results (dashed lines; areas shown in light pink indicate the standard deviation of three independent experiments) and simulation results with the estimated reaction rates based on the experimental results (solid line) are discussed in the following section. All information about the parameter estimation is summarized in Supporting Information Text S1.

**Figure 3:**
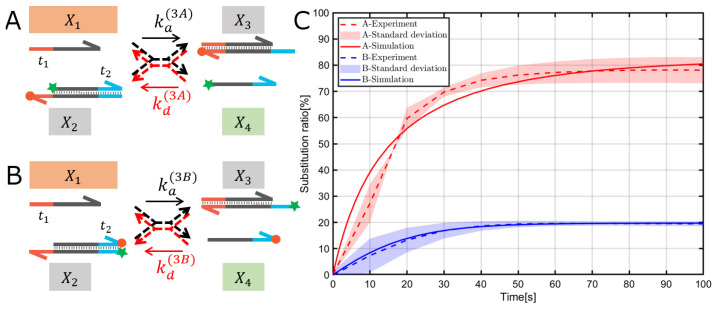
Reversible TMSDRs with the 3′ toehold case: TMSDRs with the asymmetric modification (**A**) and symmetric modification (**B**) were designed where the star and round shapes denote FAM and BHQ1, respectively. Reaction rate constants of the forward and backward reactions are denoted by $k_{a}^{\left(*\right)}$ and $k_{d}^{\left(*\right)}$ (${\mathrm{nM}}^{-1}s^{-1}$), respectively. (**C**) Experimental results (dashed lines; areas shown in light pink indicate the standard deviation of three independent experiments) and simulation results with the estimated reaction rates based on the experimental results (solid line). All information about the parameter estimation is summarized in Supporting Information Text S1.

Fluorescence measurements were performed using FP-8300 (JASCO, Corp., Tokyo, Japan) with a quartz microcell. Fluorescence spectra were taken using FAM; hence, the excitation wavelength was 495 nm, and the emission wavelength was 520 nm. For all fluorescence measurements, a 5 nm slit was used for both the excitation and emission monochromators. Experiments were performed with an integration time of 1 s for every 10 s, for a total of 90 min. To initiate the reaction, samples were prepared in a quartz microcell with a stirrer, a gate, and TE/${\mathrm{Mg}}^{2+}$ buffer containing TWEEN20 (Sigma-Aldrich Co. LLC, Burlington, MA, USA) at a concentration of 0.01%. The signal and sample temperature were allowed to stabilize for at least 120 s before the reaction began. The quartz microcell was thoroughly washed with Milli-Q purified water and 70% ethanol and dried completely for the next experiment. The experiment was performed thrice per reaction system, and the mean and standard deviation were obtained. In all cases, the fluorescence of $X_{2}$ in Figure 2 and Figure 3 were used as the baseline because both the fluorescent and quenched molecules were modified, and therefore, no reaction occurred. The fluorescence of $X_{4}$ in Figure 2A and Figure 3A and $X_{3}$ in Figure 2B and Figure 3B were used as a positive control to indicate a substitution ratio of 100% because only the fluorescent molecules are modified.

## 3. Results

### 3.1. Reversible TMSDR with the 5′ Toehold Case

The reversible TMSDRs with the 5′ toehold were investigated: the fluorophore dye (FAM) and quencher (BHQ1) were placed close together on the gate $X_{2}$ to maximize the FRET efficiency (Figure 2A), which is called symmetric modification in this paper. In contrast, the reporter molecules were placed at a distance of six mers apart through the toehold domain $t_{1}$ on the gate $X_{2}$ (Figure 2B), which is also called an asymmetric modification. For the reversible TMSDRs, the transient and steady-state characteristics are determined mainly by the length and C/G content of the toehold domains $t_{1}$ and $t_{2}$ and the thermodynamic stability of the gates $X_{2}$ and $X_{3}$. We designed the base sequences to have almost 50% of substitution ratio of $X_{1}$ strand, which is defined by the ratio of the concentration of $X_{1}$ after the reaction to that before the reaction to facilitate the discussion from the experimental data. The length and C/G content of the toehold domains and the base sequences of the recognition domain were determined using the NUPACK analysis.

As shown in Figure 2C, in the case of symmetric modification, the substitution ratio was roughly 10%, indicating that the displacement reaction starting at domain $t_{2}$ (backward reaction) seemed much more dominant than that starting at domain $t_{1}$ (forward reaction). Meanwhile, in the case of asymmetric modification, the substitution ratio was improved by roughly 30%, which was a better result than the symmetric modification case.

Therefore, the reporter modification considerably influenced the steady-state characteristics of the reversible TMSDRs, and the asymmetric modification of the reporter molecules showed a relatively lower impact on the dynamics of the reversible TMSDRs.

### 3.2. Reversible TMSDR with the 3′ Toehold Case

The reversible TMSDRs with the 3′ toehold were investigated: Figure 3A corresponds to TMSDR with the asymmetric modification in which the fluorophore dye (FAM) and quencher (BHQ1) were placed at a distance of 6 mers apart through the toehold domain $t_{1}$ on the gate $X_{2}$. Meanwhile, Figure 3B corresponds to TMSDR with the symmetric modification in which the reporter molecules were placed close together on the gate $X_{2}$. Moreover, we designed the base sequences of the TMSDRs to have nearly 50% of substitution ratios of $X_{1}$ strand as well as the 5′ toehold case.

As shown in Figure 3C, in the case of symmetric modification, the substitution ratio was roughly 20%, indicating that the displacement reaction starting at domain $t_{2}$ (backward reaction) was considerably more dominant than that starting at domain $t_{1}$ (forward reaction). Meanwhile, in the case of asymmetric modification, the substitution ratio was roughly 80%, indicating that the displacement reaction starting at domain $t_{1}$ (forward reaction) was considerably more dominant than that starting at domain $t_{2}$ (backward reaction).

Therefore, the reporter modification considerably influenced the steady-state characteristics of the reversible TMSDRs, and the symmetric and asymmetric modifications of the reporter molecules showed the opposite impact on the dynamics of TMSDRs.

### 3.3. Quantitative Analysis of the Reaction Rate Constants of the Reversible TMSDRs

The mathematical models of the TMSDRs with 5′ and 3′ toeholds share an identical structure, differing only in the reaction rate constants (Supporting Information Text S1). Therefore, comparing the reaction rate constants to clarify how the reporter modifications influenced the dynamics of the TMSDRs in both cases is warranted. Table 1 shows the reaction rate constants estimated using the experimental data (Supporting Information Text S1).

We estimated two parameters, $k_{a}^{\left(*\right)}$ and $k_{d}^{\left(*\right)}$, simultaneously for each condition and succeeded in estimating parameters that fit the experimental data well, as shown in Figure 2C and Figure 3C. Initially, we expected that the reaction rate constants would be close to the theoretical values in the absence of modification of the reporter molecules near the toehold domain. In fact, regarding the TMSDR with 5′ toehold case, the rates $k_{a}^{\left(2A\right)}$ and $k_{d}^{\left(2B\right)}$ in Figure 2A,B were reasonably close to the theoretical values as shown in Table 1. Meanwhile, regarding the TMSDR with the 3′ toehold case, the reaction rates $k_{d}^{\left(3A\right)}$ and $k_{a}^{\left(3B\right)}$ were also expected to be close to the theoretical values, yet the results deviated significantly from the theoretical values as shown in Table 1. Although we performed parameter estimation of $k_{a}^{\left(3A\right)}$ and $k_{d}^{\left(3B\right)}$ while fixing the reaction rates $k_{d}^{\left(3A\right)}$ and $k_{a}^{\left(3B\right)}$ to theoretical values, elucidating the corresponding experimental data with the estimated parameters was impossible (Supporting Information Text S4). Therefore, we conclude that the estimated results obtained in Table 1 were reasonable.

## 4. Discussion

Reporter molecules modified at toehold domains of TMSDRs have complexly acted on the reaction systems [7]. In our study, the four variations of reversible TMSDRs were investigated, where the symmetric and asymmetric modifications of the reporter molecules at 5′ and 3′ toehold were designed.

Regarding the reversible TMSDR in the 5′ toehold case along with the symmetric modification (Figure 2A), the forward reaction $k_{a}^{\left(2A\right)}$ was hardly affected by the reporter molecules because no modifications were observed at approximately the 5′ toehold as a binding site. According to [7], the BHQ1-modified lower strand of the gate $X_{3}$, which includes the binding site for the backward reaction, can accelerate the displacement reaction, therefore possibly affecting the increase of the reaction rate constant $k_{d}^{\left(2A\right)}$. This speculation considerably agreed with our experimental results (first row in Table 1).

Regarding the reversible TMSDR in the 5′ toehold case along with the asymmetric modification (Figure 2B), the backward reaction $k_{d}^{\left(2B\right)}$ is hardly affected by the reporter molecules because no modifications were observed at approximately the 3′ toehold as a binding site. Meanwhile, the BHQ1-modified upper and the FAM-modified lower strands of the gate $X_{2}$, which includes the binding site for the forward reaction, can decelerate and accelerate the displacement reaction, respectively, according to [7]. Although these conflicting effects of the modification of reporter molecules were complex to predict, the estimated rate constants implied an effect to slightly decrease the reaction rate constant $k_{a}^{\left(2B\right)}$ while $k_{d}^{\left(2B\right)}$ was reasonably close to the theoretical value (second row in Table 1). Collectively, the asymmetric modification of the reporter molecules might be preferable to the symmetric modification.

Regarding the reversible TMSDR in the 3′ toehold case along with the asymmetric modification (Figure 3A), the FAM-modified upper and the BHQ1-modified lower strands of the gate $X_{2}$, which includes the binding site for the forward reaction, can accelerate the displacement reaction according to [7]. In contrast, the backward reaction $k_{d}^{\left(3A\right)}$ was anticipated to be hardly affected by the reporter molecules because no modifications were observed at approximately the 5′ toehold as a binding site. However, the estimated $k_{d}^{\left(3A\right)}$ was unexpectedly shifted from the theoretical value. In contrast, the estimated rate constants implied an effect to slightly increase the reaction rate constant $k_{a}^{\left(3A\right)}$ as a result of conflicting effects of the modification of reporter molecules (third row in Table 1).

Finally, regarding the reversible TMSDR in the 3′ toehold case along with the symmetric modification (Figure 3B), the forward reaction $k_{a}^{\left(3B\right)}$ is hardly affected by the reporter molecules because of no modifications at approximately the 3′ toehold as a binding site. However, as in the asymmetric case above, the estimated $k_{a}^{\left(3B\right)}$ unexpectedly shifted from the theoretical values. The FAM-modified lower strand of the gate X3, which includes the binding site for the backward reaction, can accelerate the displacement reaction and, therefore, possibly affect the increase of the reaction rate constant $k_{d}^{\left(3B\right)}$. This speculation considerably agreed with our experimental results (fourth row in Table 1).

In conclusion, the reversible TMSDR in the 5′ toehold case, along with the asymmetric modification shown in Figure 2B, might be preferred for the design of the DNA reaction system and its validation experiments in terms of less modification effect while guaranteeing the FRET efficiency. On the other hand, the mechanism by which the reaction rates $k_{d}^{\left(3A\right)}$ and $k_{a}^{\left(3B\right)}$ shift from the theoretical values is unknown and requires further study. In this study, we focused on a specific toehold length (6 mers) to determine if the position of fluorophore labels affects the TMSDR. By concentrating on a specific toehold length, we aimed to isolate and analyze the behaviors and interactions within the TMSDR mechanism without introducing the complexity of varying toehold lengths. This controlled approach allowed us to measure the influence of label positions precisely. Future research should focus on increasing the diversity of toehold lengths and different base compositions with the use of other dyes and quenchers, like Alexa and Cy5, to comprehensively understand their influence on reaction kinetics.

## 5. Conclusions

In this study, we investigated the effects of reporter molecule modifications on the reaction dynamics of reversible toehold-mediated strand displacement reactions, which are integral to DNA chemical reaction networks. While previous studies have demonstrated that such modifications influence the transient characteristics of irreversible TMSDRs, our results show that in reversible systems, the impact extends to both transient and steady-state characteristics. This finding is crucial for the rational design of DNA circuits, as these circuits often incorporate both reversible and irreversible reactions.

We demonstrated that the modification of fluorophore dye and quencher can significantly tune reaction dynamics. However, by modifying the fluorescent dye and quencher an appropriate distance apart, we can mitigate adverse effects on the system while still enabling effective fluorescence resonance energy transfer measurements. These insights are vital for the accurate design and verification of DNA-based circuits, specifically in scenarios where complex information processing is required.

In summary, our results highlight the importance of careful consideration of reporter molecule modifications during the design phase of DNA reaction systems. Future work should focus on further quantifying these effects across a broader range of reaction conditions and exploring potential optimization strategies for minimizing disruption to reaction kinetics.

## Figures and Tables

**Table 1:** Estimation of reaction rate constants of the reversible TMSDRs. The ratios of the reaction rate constant to the theoretical value, $k_{a}^{\left(*\right)}=k_{d}^{\left(*\right)}=5.0\times 10^{-4}\left({\mathrm{nM}}^{-1}s^{-1}\right)$ calculated based on the toehold length of 6 mers according to [5] are shown in parentheses.

Toeholds	Modifications	Experiments	$k_{a}$ [${\mathrm{nM}}^{-1}s^{-1}$]	$k_{a}/k_{a}^{\left(*\right)}$	$k_{d}$ [${\mathrm{nM}}^{-1}s^{-1}$]	$k_{d}/k_{d}^{\left(*\right)}$
5′ toehold	symmetric	Figure 2A	$4.78\times 10^{-5}$	0.096	$4.60\times 10^{-3}$	9.2
5′ toehold	asymmetric	Figure 2B	$1.74\times 10^{-4}$	0.35	$8.51\times 10^{-4}$	1.7
3′ toehold	asymmetric	Figure 3A	$6.48\times 10^{-4}$	1.30	$2.98\times 10^{-5}$	0.060
3′ toehold	symmetric	Figure 3B	$9.68\times 10^{-5}$	0.19	$1.60\times 10^{-3}$	3.2

## Data Availability

The source code and research data is available from Git hub (https://github.com/toyomasa11/Quantitative-Analysiss-of-the-Effect-of-Fluorescent-Labels-code).

## Supplementary Materials

The following supporting information can be downloaded at: https://www.mdpi.com/article/10.3390/mi15121466/s1, Supporting Text S1: Mathematical model of TMSDRs and the parameter estimation method, Supporting Text S2: Base sequences, Supporting Text S3: NUPACK analysis, Supporting Text S4: One-parameter fitting based on theoretical values.

## Abbreviations

The following abbreviations are used in this manuscript:

DNA	Deoxyribonucleic acid
TMDSR	Toehold-mediated strand displacement reaction
FRET	Fluorescence resonance energy transfer
FAM	Fluorescein
BHQ	Black hole quencher
