# Supplementary material for: Quantitative Analysis of the Effect of Fluorescent Labels on DNA Strand Displacement Reaction
Source: Micromachines (Basel). 2024 Nov 30;15(12):1466. doi: 10.3390/mi15121466 (PMC11676983; doi:10.3390/mi15121466)
Supplement: Supplementary file 1 [file micromachines-15-01466-s001.zip › micromachines-3256967-supplementary.pdf]

・Supporting Information

Text S1. TMSDR mathematical model and the parameter estimation method

The reversible TMSDR mathematical model (Figure 2-3) is described by ordinary differential equations based on chemical kinetics, as described below:

$$\begin{cases} \frac{dx_1}{dt} = -k_a x_1 x_2 + k_d x_3 x_4 \\ \frac{dx_2}{dt} = -k_a x_1 x_2 + k_d x_3 x_4 \\ \frac{dx_3}{dt} = k_a x_1 x_2 - k_d x_3 x_4 \\ \frac{dx_4}{dt} = k_a x_1 x_2 - k_d x_3 x_4 \end{cases} \quad (1)$$

where x_i denotes the concentration of strand X_i (i=1,2,3,4), and k_a and k_d are the reaction rate constants of the forward and backward reactions, respectively.

Regarding mathematical modeling, the differential equations of the reversible TMSDRs with the 5' and 3' toehold, along with the symmetric and asymmetric modifications of reporter molecules have the same structure of the right-hand sides and differ only in their parameters. Therefore, we further explain how to estimate the parameters k_a and k_d using experimental data in our study.

First, a normalization of raw data (denoted by $y_n(t)$ here), which are time series data of fluorescence intensity measured using a fluorescence spectrophotometer, was applied before the main process of the parameter estimation as follows:

$$y_n(\mathrm{t}) = \frac{y(t) - n(t)}{p(t) - n(t)} \times 100$$

where $y_n(t)$ is the normalized signal intensity $y(t)$ at a certain time t, $n(t)$ and $p(t)$ are time series data of fluorescence intensity of negative and positive control, respectively. Notably, the negative and positive control values were regarded as 0% and 100% of the substitution ratio, respectively, which were defined by the ration of the concentration of X_1 after the reaction to that before the reaction. Therefore, $y_n(t)$ is the substitution ratio of the reversible TMSDRs.

A genetic algorithm was then used to solve the following optimization problem to estimate the rate constants k_a and k_d. Genetic algorithms are optimization methods that mimic the biological evolution process and effectively optimize complex nonlinear problems. In our study, the Global Optimization Toolbox in matlab (Mathworks, Inc.) was used. The evaluation function to minimize was defined as follows:

$$\mathrm{J} = \sum_{i=1}^{n} (y_s(t_i) - y_n(t_i))^2$$

where the experimental data comprised a set of discretely sampled data points $y_n(t_i)$, where t_i $(i = 1, \dots, n)$ is the time when the data were measured. The simulation data also comprised a set of discretely sampled data points $y_s(t_i)$ from the corresponding simulation data that were calculated using the Eq. (1) with matlab solver, ode15s. The source code is available from Git hub .

Text S2. Base sequences

The base sequences designed for our experiments are listed in the following tables. The naming convention for the following sequences indicates that the initial "X_i (i=1,2,3,4)" refers to single-stranded or double-stranded DNA in Figure 2-3, the subsequent "5" and "3" denote the presence of a 5' toehold or a 3' toehold, the following "up" and "down" distinguish whether the single strand is the upper or lower strand of the double-stranded DNA, and the final "A" and "B" indicate which scheme in Figures 2-3 the DNA strand is positioned in.

Table S1. Base sequences （reversible TMSDR with the 5' toehold case）

Strand	Sequence
X_1-5	CTCCACACTACATCTCTTCCTACTCG
X_2-5-up-A	FAM-GTCTCACTCCACACTACATCTCTTCC
X_2-5-down-A	CGAGTAGGAAGAGATGTAGTGTGGAGTGAGAC-BHQ
X_2-5-up-B	GTCTCACTCCACACTACATCTCTTCC-BHQ
X_2-5-down-B	FAM-CGAGTAGGAAGAGATGTAGTGTGGAGTGAGAC

Table S2. Base sequences (reversible TMSDR with the 3' toehold case)

Strand	Sequence
X_1-3	GCTCATCCTTCTCTACATCACACCTC
X_2-3-up-A	FAM-CCTTCTCTACATCACACCTCACTCTG
X_2-3-down-A	CAGAGTGAGGTGTGATGTAGAGAAGGATGAGC-BHQ
X_2-3-up-B	CCTTCTCTACATCACACCTCACTCTG-BHQ
X_2-3-down-B	FAM-CAGAGTGAGGTGTGATGTAGAGAAGGATGAGC

Text S3. NUPACK analysis

All base sequences designed above were analyzed to avoid undesirable secondary structures. The DNA structure prediction by NUPACK[1] was based on the following parameters: temperature, 25°C; Na+ concentration, 50 mM; and maximum complex size, 6 strands. The results are illustrated as follows:

Figure S1. The nucleotide sequence structure corresponding to the reaction scheme in Figure 2

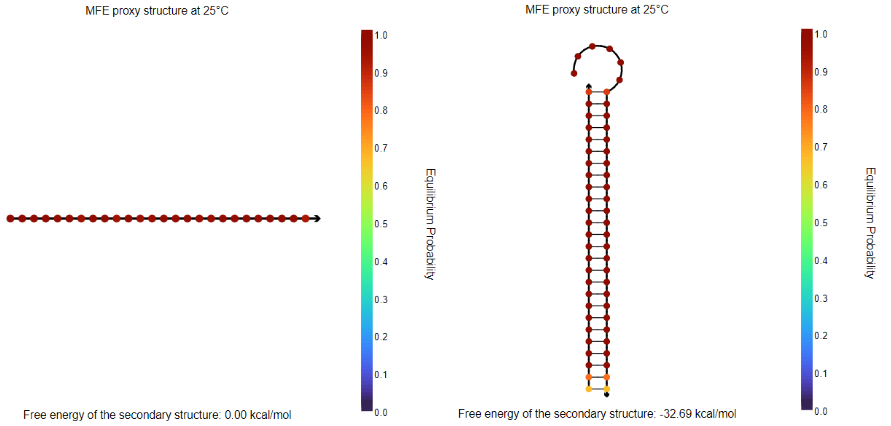

(a) X_1-5 (b) X_2-5

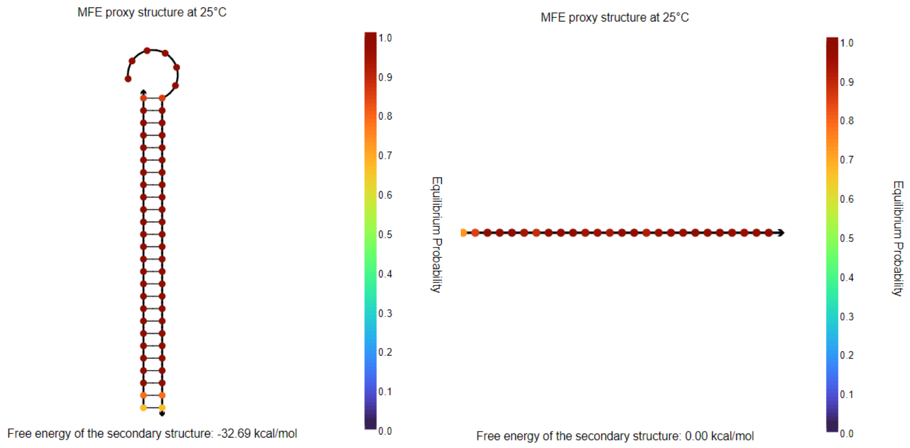

(c) X_3-5 (d) X_4-5

Figure S2. The nucleotide sequence structure corresponding to the reaction scheme in Figure 3

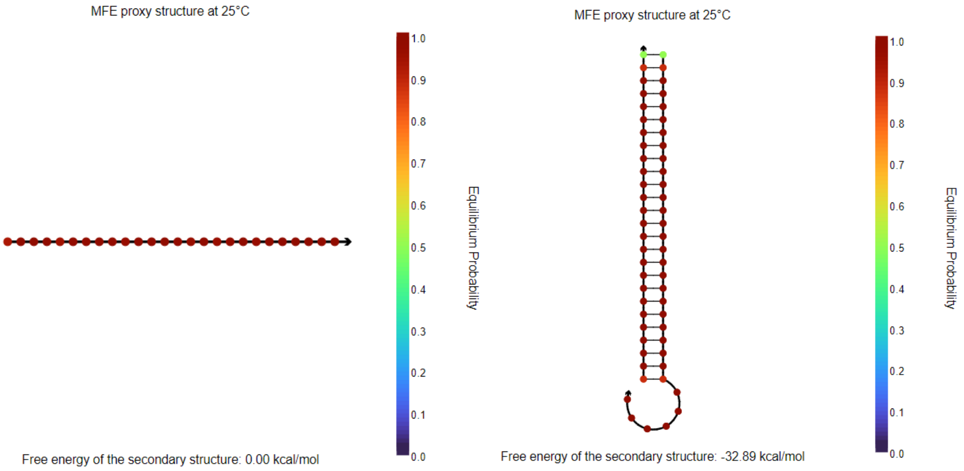

(a) X_1-3 (b) X_2-3

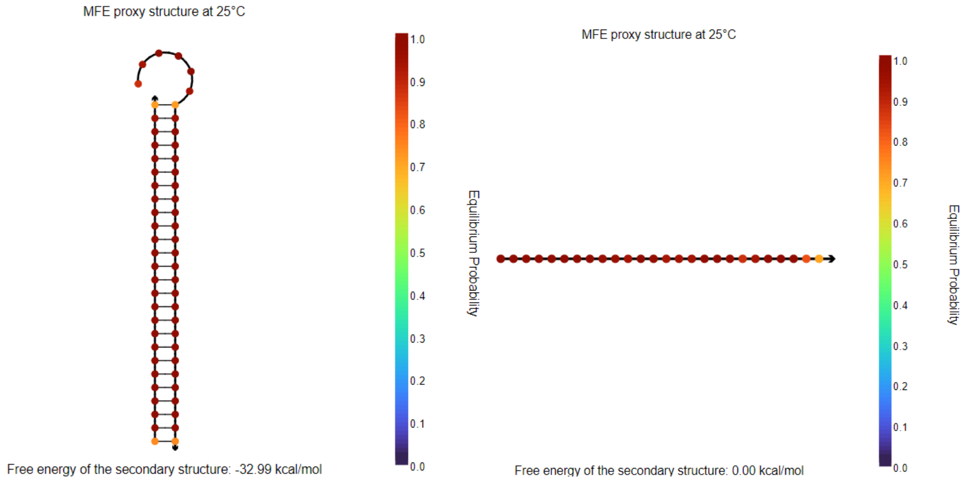

(c) X_3-3 (d) X_4-3

Text S4. Theoretical value-based one-parameter fitting

The theoretical reaction rates for the TMSDRs regarding the toehold domains that were not modified with

the reporter molecules were fixed during parameter estimation. Specifically, we fixed "k_a" and "k_d" and adopted the following approach to estimate only the remaining parameters. Reaction rate constants of the forward reactions "k_a" was fixed at its theoretical value, and only reaction rate constants of the backward reactions "k_d" was estimated in Figure S3A and S4B. "k_d" was fixed at its theoretical value and to estimate other parameters, we fixed "k_a" at its theoretical value and performed fitting in Figure. S3B and S4A.

Figure S3. Experimental (dashed lines; standard deviation of three independent experiments indicated in light pink shading) and simulation results with the estimated reaction rates based on the experimental results (solid line). The red dashed and solid line A shows the results of the reaction scheme in Figure 2A, and the blue dashed and solid line B shows the results of the reaction scheme in Figure 2B.

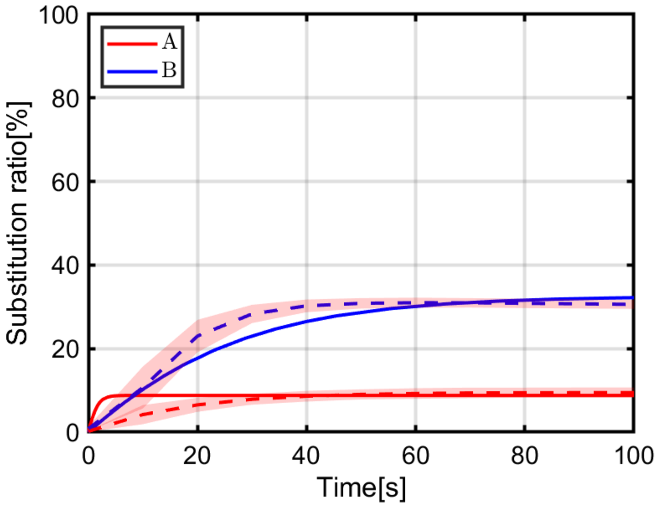

Figure S4. Experimental results (dashed lines; standard deviation of three independent experiments indicated in light pink shading) and simulation results with the estimated reaction rates based on the experimental results (solid line). The red dashed and solid line A shows the results of the reaction scheme in Figure 3A, and the blue dashed and solid line B shows the results of the reaction scheme in Figure 3B.

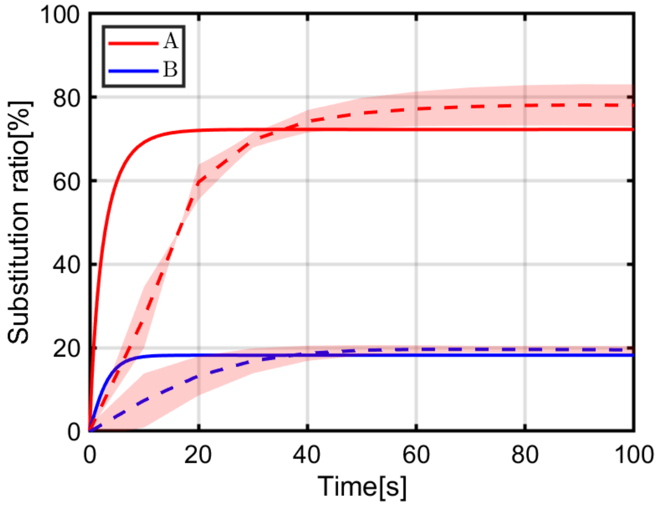

Table S3. Reaction rate constant estimation of the reversible TMSDRs. The ratios of the reaction rate constant to the theoretical value, $k_a^* = k_d^* = 5.0 \times 10^{-4}$nM^{-1}s^{-1} calculated based on the toehold length of 6 mers are shown in parentheses.

toeholds	modifications	experiments	k_a	k_a/k_a^*	k_d	k_d/k_d^*
5'toehold	symmetric	Figure S3A	5.0×10^{-4}	1	5.3×10^{-2}	106
5'toehold	asymmetric	Figure S3B	1.1×10^{-4}	0.22	5.0×10^{-4}	1
3'toehold	asymmetric	Figure S4A	3.4×10^{-3}	6.8	5.0×10^{-4}	1
3'toehold	symmetric	Figure S4B	5.0×10^{-4}	1	1.0×10^{-2}	20

・Reference

[1] J. N. Zadeh, C. D. Steenberg, J. S. Bois, B. R. Wolfe, M. B. Pierce, A. R. Khan, R. M. Dirks, and N. A. Pierce. NUPACK: Analysis and design of nucleic acid systems. Journal of Computational Chemistry, 32:170–173, 2010.
